# Shrub‐facilitated invasion accelerates desertification

**DOI:** 10.1002/eap.70162

**Published:** 2025-12-08

**Authors:** Jacob E. Lucero, Christopher J. Lortie, Alessandro Filazzola, Ragan M. Callaway

**Affiliations:** ^1^ Department of Rangeland, Wildlife and Fisheries Management Texas A&M University College Station Texas USA; ^2^ Department of Biology York University Toronto Ontario Canada; ^3^ Apex Resource Management Solutions Ottawa Ontario Canada; ^4^ Division of Biological Sciences University of Montana Missoula Montana USA

**Keywords:** aboveground net productivity, biodiversity, desertification, facilitated invasion, facilitation, fire, invasion, Mojave Desert, precipitation marginal response, precipitation use efficiency

## Abstract

In theory, increasing sensitivity of primary productivity to precipitation variability is a biophysical symptom of dryland degradation, or “desertification,” but empirical tests of this in the context of biological invasions are scant. To test the potential for exotic grass invasion to exacerbate biophysical symptoms of desertification, we measured the biomass and biodiversity of herbaceous plant assemblages along a 41–248 mm/year precipitation gradient across the Mojave and San Joaquin Deserts within communities at high versus low levels of exotic grass invasion and under shrub canopies versus interstitial space, over 5 years. Exotic grass invasion doubled the conversion rate of precipitation into biomass, and native shrubs increased ecosystem sensitivity to precipitation through strong facilitation of exotic grasses. Invasion‐driven increases in biomass production corresponded to significant decreases in native biodiversity. We propose that shrub facilitation of exotic grasses accelerated desertification by promoting a non‐native flora that is highly sensitive to precipitation variability and strongly linked to biodiversity degradation. Suppressing exotic grasses and managing facilitated invasion will help mitigate desertification.

## INTRODUCTION

Desertification destabilizes ecological communities and human societies in drylands across the globe (Reynolds et al., 2007). Desertification can be caused by many interacting factors including climate variability and anthropogenic disruption, and biophysical aspects of desertification are often expressed through changes in biological productivity or ecological integrity in response to environmental change (Verón et al., 2006). In this context, theoreticians have proposed two key biophysical measurements: the precipitation marginal response (PMR), which focuses on the sensitivity of primary productivity to annual variation in precipitation; and precipitation use efficiency (PUE), which defines the capacity of an ecosystem to convert water into growth or biomass (Verón et al., 2018; Verón & Paruelo, 2010). For these measurements, high sensitivity to variation in precipitation or a poor capacity to convert water into biomass reflects ecosystem‐based symptoms of desertification. However, despite the potential for precipitation‐productivity relationships to diagnose desertification, we lack field‐based tests. This limits our ability to respond to the social, ecological, and economic threats of desertification (Verón et al., 2006).

Exotic grass invasions interact with altered precipitation patterns associated with climate change (Ravi et al., 2022) and thus provide important opportunities to examine nuanced biophysical symptoms of biotic and abiotic drivers of desertification. These invasions can be powerful biotic drivers of desertification (Ravi et al., 2009) because of their pervasive and profound negative impacts on native biodiversity and ecosystem functioning across very large spatial and temporal gradients of annual precipitation (Lortie et al., 2024; Lucero et al., 2020, 2021). However, the biophysical connection of exotic grass invasion to desertification can be complex and, in some respects, counterintuitive. For example, exotic plant species commonly increase mean annual net primary productivity (ANPP) by replacing smaller statured native species or filling in previously bare ground (Liao et al., 2008). In addition to increasing ANPP, exotic grass invasion of desert shrublands can increase PMR when exotic invaders display a highly plastic response to precipitation variability, and PUE when exotic invaders have relatively rapid growth rates and so capitalize on precipitation events more quickly than native perennials (Ravi et al., 2009). Together, a simultaneous increase in ANPP, PMR, and PUE in invaded sites relative to non‐invaded sites, concomitant with reductions in native biodiversity, would be symptomatic of desertification via exotic plant invasion (Verón et al., 2006), though this theory has never been tested. Furthermore, the intensity of annual grass invasion in deserts can be strongly affected by the presence of native shrubs, with the abundance of exotic grasses often many times higher under‐shrub canopies than in interstitial space (Abella & Smith, 2013; Lucero et al., 2020), corresponding to more intense negative impacts on native species under shrubs than out in the open (Lucero et al., 2021). Such “facilitated invasion” by shrubs across climatic and soil fertility gradients (Lortie et al., 2022; Schlesinger et al., 1996) collectively provides an opportunity to explore novel biophysical axes of desertification driven by exotic plant invasion.

PMR and PUE have been widely used to evaluate biophysical symptoms of grassland degradation (Verón et al., 2018; Verón & Paruelo, 2010), but they have not been used to quantify biophysical symptoms of desertification via exotic plant invasion, much less desertification via shrub‐facilitated invasion. Importantly, quantification of PMR and PUE in the context of land degradation has been limited to remotely sensed estimates or proxies for ANPP and not measured directly in the field, and never in the context of exotic invasion—with or without native shrub facilitation. Here, we combine field sampling of ANPP over five consecutive years across the Mojave and San Joaquin Deserts along substantial temporal and spatial precipitation gradients, along an extensive invasion gradient, and in the presence versus absence of native shrubs to explore the potential for exotic grass invasion—primarily by *Bromus madritensis* subsp. *rubens* and *Schismus* species—and shrub facilitation to influence relationships among ANPP, relative water availability, and plant biodiversity. Since exotic plant invasions generally increase ANPP (Liao et al., 2008), we predicted that increasing plant invasion would increase PMR and PUE at all levels of annual precipitation, sensu Verón et al. (2018). Given that desert shrubs commonly and strongly facilitate invasive grasses (Lortie et al., 2024; Lucero et al., 2020, 2021), we further predicted that shrubs would exaggerate invasion‐driven ANPP, PMR, and PUE and hence indirectly accelerate desertification through facilitated invasion across our precipitation gradient. Facilitated invasion by shrubs will occur most frequently at the mesic end of our precipitation gradient (Lucero, Faist, et al., 2022; Lucero, Filazzola, et al., 2022) if increasing precipitation activates the disproportional uptake of nutrients under shrubs by exotic species (Scholes, 1990). As invasive species commonly exclude natives, we also predicted that increasing abundance of exotics and their associated contribution to ANPP would suppress native abundance and diversity at all points on the climatic gradient.

## METHODS

### Study system

We used a dataset concatenated from five independent and previously published studies (Lortie et al., 2024; Lucero et al., 2020, 2024; Lucero, Faist, et al., 2022; Lucero, Filazzola, et al., 2022) that surveyed annual plant communities between 2016 and 2020 at peak flowering in March and April at 22 sites (Appendix S1: Figure S1) that spanned a precipitation gradient of 41 to 248 mm/year across the Mojave and San Joaquin Deserts, USA. Primary data have been publicly archived (Filazzola et al., 2020a, 2020b; Lortie, 2024; Lucero et al., 2024). We selected sites in climax native shrub communities. Sites were screened regionally using remote sensing and field surveys to ensure independence and representativeness of the plant communities within the region.

Sites in the Mojave Desert were dominated primarily by the native shrub *Larrea tridentata*, representing >95% of the shrubs sampled, but we also sampled very low numbers under the native perennials *Ambrosia dumosa* and *Lycium andersonii*. Sites in the San Joaquin Desert were dominated by the native shrub *Ephedra californica*, which was the only shrub we sampled there, but the native perennials *Atriplex lentiformis, Ericameria linearifolia*, and *Agave americana* were present at much lower densities. All sites were invaded to some degree by the exotic annual grasses *B. madritensis* and *Schismus* spp., although these invaders were not present in all plots. Among the exotic species present, we quantified only *B. madritensis* and *Schismus* spp. because they were >95% of the invasive abundance, and they are among the region's most problematic invasive species (Salo, 2004) due to strong negative impacts on community‐level biodiversity (Salo, 2004) and fire cycles (Fusco et al., 2019). During the study, annual precipitation at the study sites ranged from 41 to 248 mm/year. Climate data were collected from the National Oceanic and Atmospheric Administration (NOAA) database, with some supplementation from local weather stations for missing data.

### Vegetation sampling

In each of the four studies, annual plant communities were sampled using paired shrub‐open microsite plots with 0.5 × 0.5 m (0.25 m^2^) quadrats subdivided into 100, 5‐cm^2^ frames. Shrub microsites were defined as the area immediately beneath the canopy of a shrub, and open microsites were defined as interstitial spaces at least 1 m from any shrub canopy. For shrub microsites, sampling quadrats were centered midway between the shrub center and dripline. We did not sample areas more than 5 m away from shrubs. Shrub‐open pairs for sampling were chosen haphazardly at each site. The original datasets contained 2040 plots, equally divided between shrub and open microsites. For each of the site/year combinations, we sorted data into four categories, under‐shrub plots with low invasion (≤20% of the total density was invasive grasses), under‐shrub plots with high invasion (>20% of the total density was invasive grasses), open plots with low invasion, and open plots with high invasion. In the 0.25‐m^2^ quadrats, we recorded the total density (number of plants rooted inside the quadrat) of *B. madritensis*, *Schismus* spp., and native species (pooled), and the richness of native species. ANPP was estimated using a 20‐cm‐diameter ring placed in the center of 840 of the plots. Biomass for the remaining plots was estimated using a regression with data from the plots in which both density and biomass were sampled and generated using the “predict” function in R (*r* ^2^ = 0.67; df = 1,839; *p* < 0.0001). We used the means of these categories in each independent site/year for our analyses. If a site/year combination had five or fewer plots in a category we discarded those data and thus lost a replicate for that category. This was most common for low‐invasion plots. We also discarded plots in which biomass was not empirically measured and with densities outside of the range of the density/biomass regression described above. Thus, the final dataset contained 1533 plots, with 819 under shrubs and 714 in the open, distributed among 44 shrub versus open and site/precipitation combinations.

### Statistical analyses

Mean ANPP was compared between the open and shrub microsites with a Mann–Whitney U test due to non‐normal data distributions. The PMR was calculated using the means of each of the four categories described above as replicates. For each of the four categories, ANPP (the dependent variable) was regressed against annual precipitation (the independent variable). PUE was calculated as ANPP/annual precipitation and regressed against annual precipitation. Mean PUEs were compared with Mann–Whitney rank sum tests because of non‐normal distributions. In shrub and open microsites, ANPP was regressed against the proportion of invasive grasses, the abundance (density) of native annuals was regressed against ANPP, and the richness of native annuals was regressed against ANPP. We fit each regression to both a linear and second‐order error distribution, with goodness of fit evaluated through difference in Akaike information criterion between models (ΔAIC). When ΔAIC ≥ |2|, the error distribution with the lowest AIC value was considered the best fit; when ΔAIC < |2|, models were considered competitive (Burnham & Anderson, 2002). All regressions were conducted in SigmaPlot 15.0, and AICs were calculated in R. We quantified the potential for autocorrelation in ANPP across study sites and years using Moran's *I*. We used the R package “spdep” with the functions “knearneigh” and “knn2nb” to create k‐nearest neighbor relationships among sites (we used *k* = 4) followed by the function “moran.mc” to compute Moran's *I* using a Monte Carlo permutation test to assess significance (Bivand, 2022).

## RESULTS AND DISCUSSION

### Curve fit and autocorrelation

A linear curve fit described our data just as well or better than a second‐order curve fit, except for ANPP × native abundance in shrub microsites (Appendix S1: Table S1). We detected spatial autocorrelation among study sites only in 2016 and 2017 (Appendix S1: Table S2), suggesting that for these years, sites with high ANPP were spatially clustered. Some spatial autocorrelation is not surprising because we sampled across a regional precipitation gradient in which proximal sites shared similar plant communities and possibly experienced the same precipitation events. Even so, for all other study years (2015, 2019, 2020), ANPP varied independently in space. We detected temporal autocorrelation at only one of 22 study sites (Appendix S1: Table S3), suggesting that all study sites functioned independently in ANPP from year to year, except one. This degree of autocorrelation is relatively minor (Schweiger et al., 2015) and does not substantially alter the inferences that follow.

### Precipitation and productivity

**FIGURE 1:**
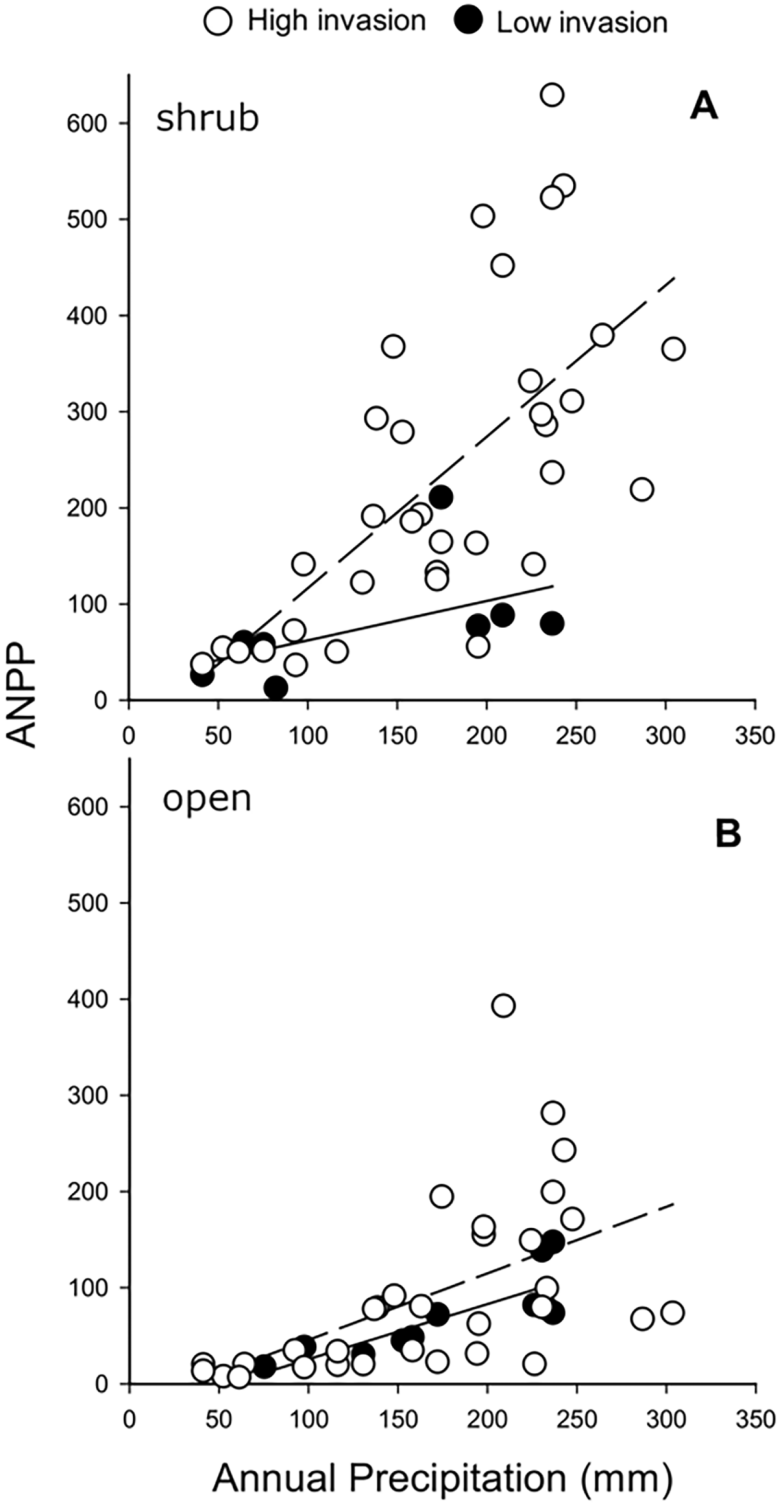
The precipitation marginal response (PMR) for herbaceous vegetation in the Mojave Desert. Open circles and dashed lines represent high‐invasion plots (>20% invasives), closed circles and solid lines represent low‐invasion plots (≤20% invasives). (A) PMRs for herbaceous vegetation under shrub canopies. For high‐invasion plots, *r* ^2^ = 0.45, *p* < 0.001, for low‐invasion plots, *p* = 0.184. (B) PMRs for herbaceous vegetation in the open. For high‐invasion plots, *r* ^2^ = 0.29, *p* = 0.001, for low‐invasion plots, *r* ^2^ = 0.67, *p* = 0.002. For slope comparisons, analysis of covariance (ANCOVA), *F*_plot type×annual precipitation_ = *F* = 0.0836, *p* = 0.774. ANPP, annual net primary productivity.

In support of both predictions, high levels of exotic grass invasion correlated with larger PMR values (i.e., exotic invasion increased the sensitivity of ANPP to precipitation variability), and this effect was far stronger under shrubs than in adjacent open microsites. Under shrubs, mean ANPP of all herbaceous plants across all microsites and years was 189.3 ± 24.5 versus 67.8 ± 12.5 g/m^2^ in open microsites adjacent to shrubs (Mann–Whitney *U* statistic = 442.0; *p* < 0.001). PMR for the herbaceous understory beneath shrub canopies was much higher for high‐invasion microsites than low‐invasion microsites (Figure 1A). Under shrubs, ANPP increased much more rapidly with annual precipitation in high‐invasion microsites. For high‐invasion plots, ANPP and annual precipitation were positively related (*r* ^2^ = 0.45; df = 1,33; *p* < 0.001), but for low‐invasion plots, they were not (*r* ^2^ = 0.27; df = 1,7; *p* = 0.184). In open microsites, ANPP and annual precipitation were positively related for both high‐ (*r* ^2^ = 0.29, df = 1,31, *p* = 0.001) and low‐invasion plots (*r* ^2^ = 0.67; df = 1,10; *p* = 0.002), but the PMRs (slopes) did not differ (Figure 1B, analysis of covariance (ANCOVA), *F*_plot type×annual precipitation_ = 0.083; df = 1,10; *p* = 0.774). Our findings confirm that dynamic changes in the way vegetation converts precipitation into ANPP (i.e., the PMR) can be a useful, field‐based method for assessing desertification (Verón et al., 2006) and provide the first application of these measures to desertification via facilitated invasion.

**FIGURE 2:**
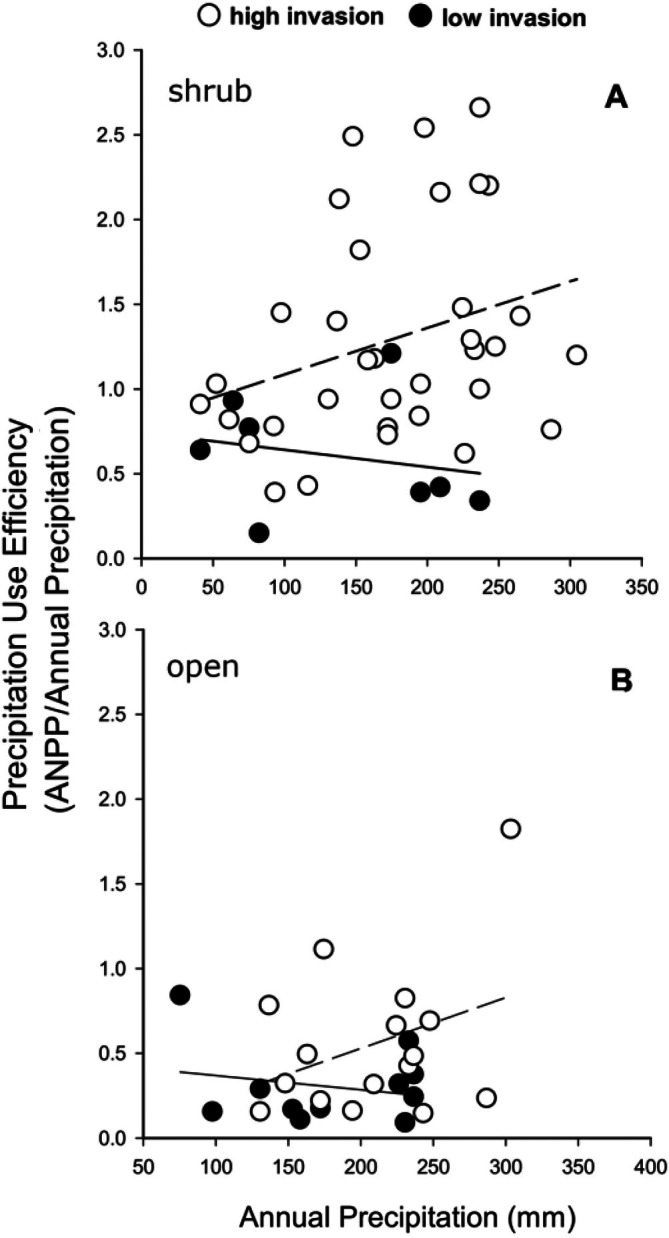
Precipitation use efficiency (PUE) for herbaceous vegetation in the Mojave Desert. Open circles and dashed lines represent high‐invasion plots (>20% invasives), closed circles and solid lines represent low‐invasion plots (≤20% invasives). (A) PUEs for herbaceous vegetation under shrub canopies. Neither regression is significant. (B) PUEs for herbaceous vegetation in the open. Neither regression is significant. ANPP, annual net primary productivity.

High‐invasion plots also had greater mean PUE values than low‐invasion plots (i.e., heavily invaded plots converted precipitation into ANPP more effectively than lightly invaded plots), but only in association with native shrubs. Mean PUE of herbaceous plants under shrubs for high‐invasion plots was 1.29 ± 0.11 versus 0.61 ± 0.12 for low‐invasion plots (Mann–Whitney rank sum test, *p* = 0.002). However, annual precipitation did not significantly predict PUE under shrubs or in the open at either high or low levels of invasion (*r* ^2^ = 0.09; df = 1,33; *p* = 0.081 and *r* ^2^ = 0.05; df = 1,7; *p* = 0.597; Figure 2A). In open microsites, there was no difference in mean PUE and no significant relationships for high‐ or low‐invasion plots (*p* > 0.25). By promoting invasion, shrubs increased the conversion of precipitation into ANPP.

#### Invasion and productivity

The PMR findings were explained primarily by the positive response of ANPP to exotic grass invasion, especially under‐shrub canopies. For herbaceous vegetation under shrubs, ANPP increased exponentially across the range of proportions of the densities of invasive plants to native plants, with a rapid transition when exotic invasive grasses comprised about 60% of the total density (Appendix S1: Figure S2A, *r* ^2^ = 0.27; *p* < 0.001). However, in the open microsites, there was no relationship between the proportion of invasive grass in a plot and ANPP (Appendix S1: Figure S2B, *p* = 0.124). This change in the PMR strongly suggests that invasive grasses acquired resources more rapidly than their native herbaceous neighbors, but the crucial role of shrubs in this conversion indicates that enhanced resource acquisition is more complex than direct responses to precipitation. We suggest that shrub effects on PMR and PUE are likely due to higher soil moisture and nutrients under shrubs than in the open—a common spatial pattern in arid shrublands (Schlesinger et al., 1996). These “fertile islands” under shrubs can substantially increase the slope of the precipitation/ANPP relationship, suggesting that desertification via exotic grass invasion is more intense under shrubs where soils are more fertile. Altogether, shrubs can increase water and nutrient supply, but this disproportionately benefits fast‐growing exotic plant species over natives (Besaw et al., 2011), causing fertile islands under‐shrub canopies to act as hotspots for invasion, thus accelerating biophysical rates of desertification captured by PMR and PUE. Once established under shrubs, invasive plant species can then spread into adjacent open spaces away from shrubs thereby increasing extent of invasion at larger scales (Ravi et al., 2009). In turn, increased fuel loads from invasion promote fire, which forms a positive feedback loop with invasion (Smith et al., 2023). This scenario is consistent with recent evidence that “fire needs annual grasses more than annual grasses need fire” (Smith et al., 2023).

Shrub encroachment in drylands, including native shrub expansion and exotic shrub invasions, can have extensive effects on a broad range of ecosystem properties and can drive other forms of desertification. In this context, our results suggest a domino effect because encroaching shrubs facilitate exotic grasses, which then displace native competitors. Shrub encroachment into dryland herbaceous communities is ongoing and predicted to accelerate with increasing temperatures and grazing (Biancari et al., 2024). Shrubs in our study were native and not reported to be expanding, but their interactions with other species is a double‐edged sword. In the absence of exotic grasses, or at low levels of grass invasion, shrubs in our system are associated with high native diversity and abundance in their understories relative to the open matrix adjacent to shrubs (Lucero et al., 2021). Without invasion, dryland shrubs can also drive complex cascades of interactions that organize native communities (Ridenour et al., 2023). On the other hand, arid‐land shrubs can facilitate many invasive species (Lortie et al., 2021; Lucero et al., 2020, 2021; Lucero, Faist, et al., 2022; Lucero, Filazzola, et al., 2022). Thus, the net effect of shrubs on local plant communities can shift from positive to negative, depending on which species are facilitated and the direction and magnitude of their ensuing interactions with neighbors. This suggests an urgent need for managing shrub‐facilitated invasions by controlling exotic invaders and promoting native species. Suppressing exotic grasses and promoting native species in shrub microsites will conserve many of the benefits of shrub facilitation on dryland plant communities and help combat desertification.

### Desertification and biodiversity

**FIGURE 3:**
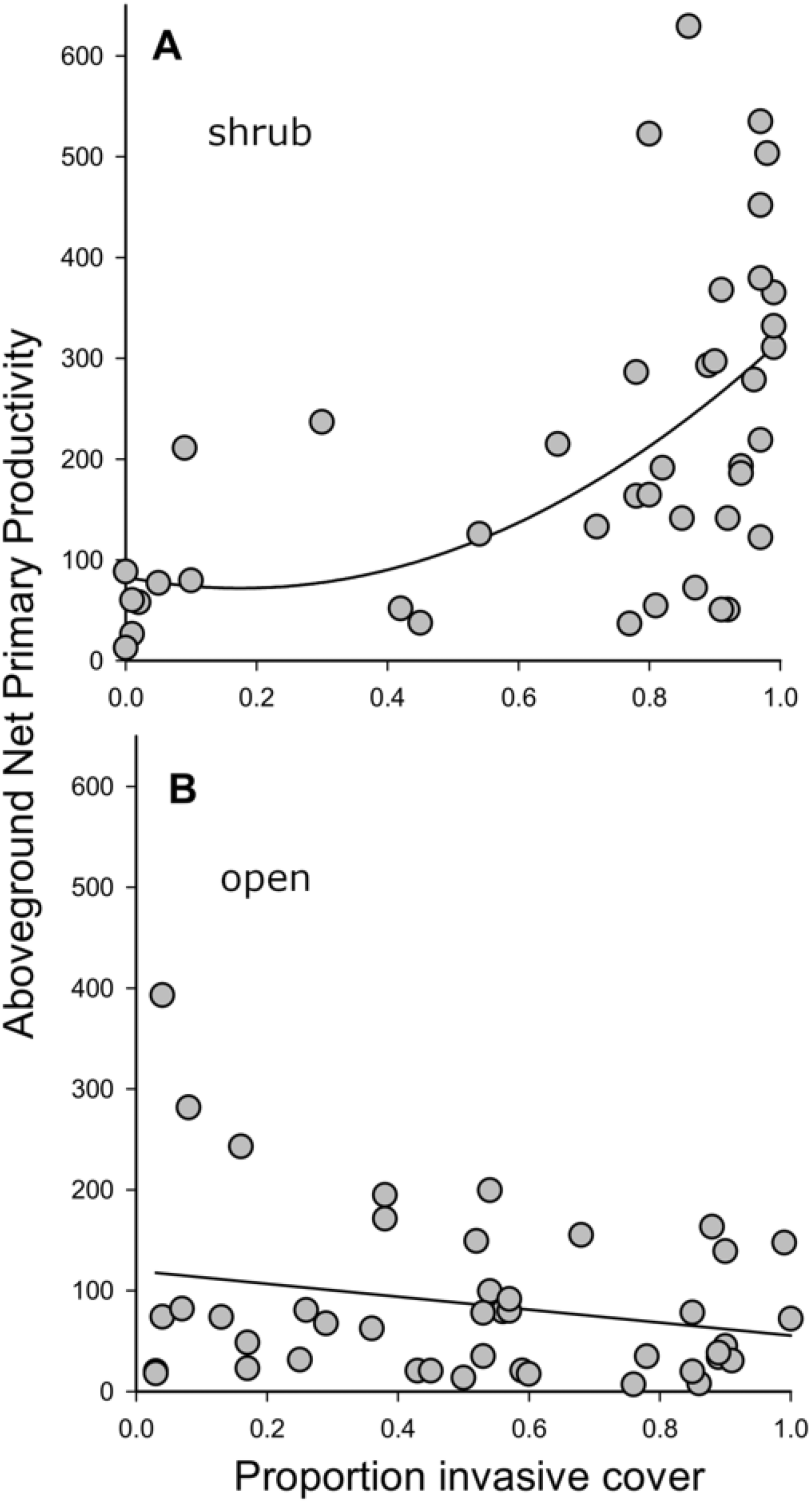
(A) The relationship between annual net primary productivity (ANPP) and native plant species abundance in shrub microsites (linear *r* ^2^ = 0.34, *p* < 0.001). (B) The relationship between ANPP and native plant species richness in shrub microsites (*r* ^2^ = 0.20, *p* = 0.003).

Another feature of desertification is biodiversity loss (Reynolds et al., 2007), and we found that increasing ANPP under shrubs, due primarily to facilitation of exotic grasses by shrubs, correlated with striking decreases in native plant density (Figure 3A, *r* ^2^ = 0.34, *p* < 0.001, Appendix S1: Table S1) and diversity (Figure 3B, *r* ^2^ = 0.20, *p* = 0.003). We suggest that highly invaded assemblages under shrubs increased local ANPP through a superior ability to convert water into biomass (i.e., relatively high PUE), compared to native assemblages (Figure 2). Relatively high PUE combined with the “fertile island” effect of shrubs likely enhanced the ability of invasive grasses to outcompete and displace native neighbors under‐shrub canopies, thereby eroding local biodiversity and ecosystem functioning (Germino et al., 2016). Importantly, increased ANPP provisioned by highly invaded assemblages under shrubs were volatile and sensitive to precipitation variability (i.e., high PMR; Figure 1). This suggests that highly invaded assemblages, especially under‐shrub canopies, may be less resilient to change than native assemblages, again a rationale for restoration with native species and managing facilitated invasion by shrubs.

### Desertification and other ecosystem changes

Our PMR measurements provide strong evidence that under shrubs, vegetation dominated by invasive grasses manifests a much more variable and dynamic response to precipitation variability than native‐dominated vegetation. This supports, to a large degree, the hypothesis of Verón et al. (2018). These changes are also supported by increased PUE in response to invasion. Related changes included the marked loss of biodiversity—a general feature of exotic plant invasion. Importantly, if desertification was evaluated in our system by changes in ANPP alone, one might incorrectly conclude the opposite, that invasive grasses “greened” the desert and moved the system away from desertification. This highlights the importance of incorporating the provenance and biodiversity impacts of species into nuanced biophysical measurements for quantifying desertification. Native biodiversity loss, a critical component of desertification (Reynolds et al., 2007), was highly associated with increasing invasion‐driven ANPP, and thus with PMR, further demonstrating that PMR is a promising on‐the‐ground diagnostic metric for invasion‐driven desertification. In this context, an interesting but untested corollary follows. If PMR can be used to diagnose desertification via exotic invasion, it can also be used to assess its reversal in response to restoration. Specifically, decreasing PMR values concomitant with decreasing invader dominance through restoration actions could suggest a reversal of desertification and hence successful restoration. We suggest the PMR can be a useful biophysical complement to biodiversity‐based metrics for evaluating restoration success, especially for projects focused on increasing ecosystem stability or decreasing volatility. Finally, our results provide strong quantitative evidence of desertification via facilitated invasion by shrubs (Ravi et al., 2009). In turn, invasion can promote fire, which in drylands often leads to permanently degraded states. The relationship between ecosystem sensitivity to precipitation, water availability, and productivity in invaded shrublands provides valuable insights into the biophysical symptoms of desertification.

## CONFLICT OF INTEREST STATEMENT

The authors declare no conflicts of interest.

## Supporting information


Appendix S1.

## Data Availability

Data sets used for this research are available in Figshare as follows: field data from vegetation survey plots (Lucero et al., 2024), https://doi.org/10.6084/m9.figshare.25989124.v2; *E. californica* and exotic grass species data (Filazzola et al., 2020a, 2020b), https://doi.org/10.6084/m9.figshare.12551087.v1; plant invasion dynamics (Filazzola & Lortie, 2024), https://doi.org/10.6084/m9.figshare.25805515.v2.
