# Supplementary material for: Shrub‐facilitated invasion accelerates desertification
Source: Ecol Appl. 2025 Dec 8;35(8):e70162. doi: 10.1002/eap.70162 (PMC12683704; doi:10.1002/eap.70162)
Supplement: Supplementary file 1 — Appendix S1. [file EAP-35-e70162-s001.pdf]

Appendix S1

Shrub-facilitated invasion accelerates desertification

Jacob E. Lucero, Christopher J. Lortie, Alessandro Filazzola, Ragan M. Callaway

Ecological Applications

Appendix S1 for this article consists of two supplementary figures (Appendix S1: Figs. S1 and S2) and three supplementary tables (Appendix S1: Tables S1–S3).

Fig. S1: Map of 22 sampling locations across the Mojave and San Joaquin Deserts of California and Nevada, USA. Mean and minimum distance between adjacent sites was 260.0 (+/- 7.4) km and 30.5 (+/- 7.5) km, respectively. Map courtesy of Google.

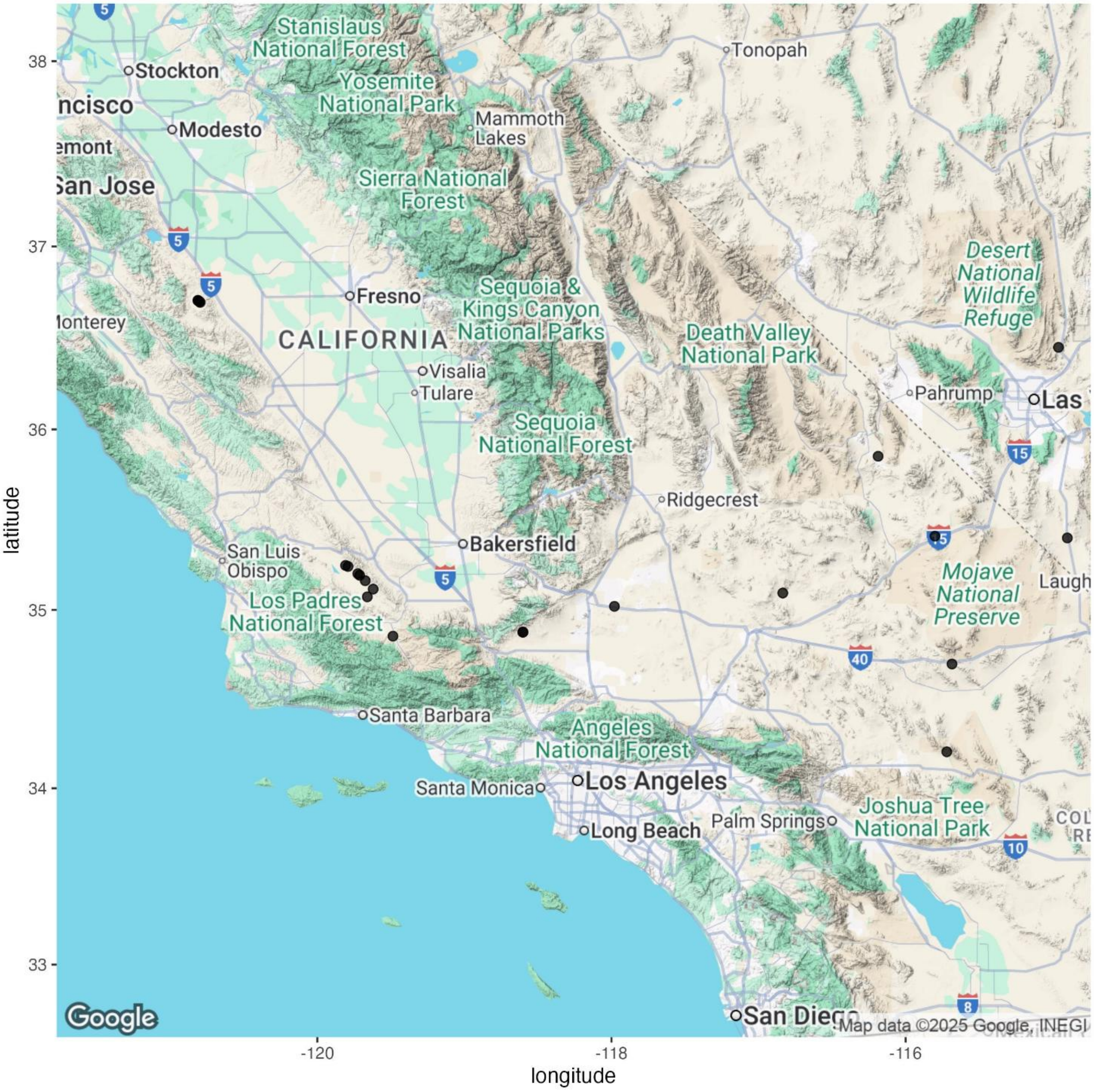

Fig. S2. Aboveground net primary productivity regressed against proportion cover by invasive annual grasses in shrub (A; $r^2 = 0.27$; $P < 0.001$) and open (B; $P = 0.124$) microsites.

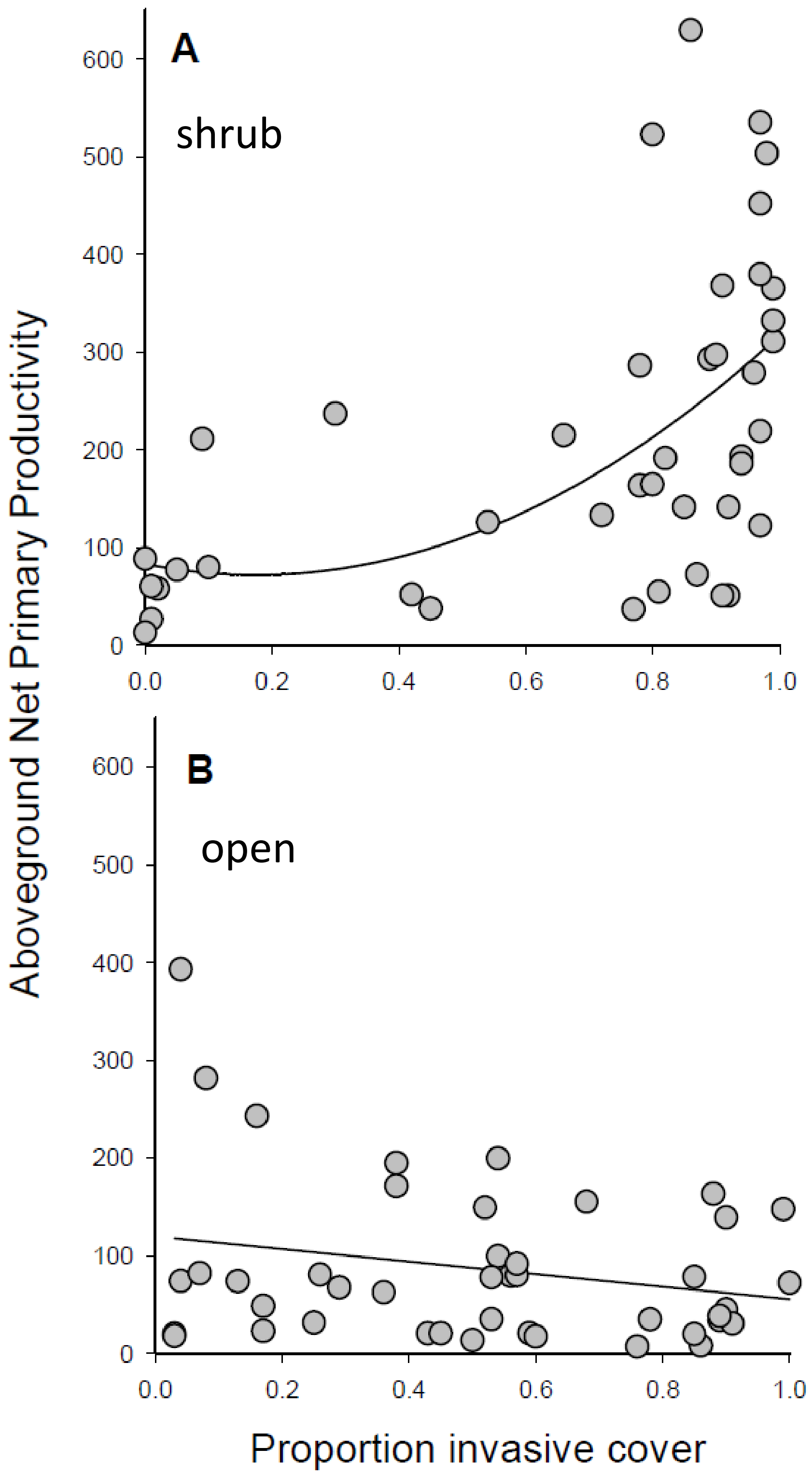

Table S1. Results of regression analyses for relationships between the tested dependent and independent variables within shrub and open microsites, and at high, low, or continuous invasion status, fit to linear or polynomial (second order; "poly") error distributions, with corresponding R^2, p- and AIC values for each model. For ΔAIC ≥ |2|, the model with the lowest AIC value is the best fit.

Tested relationship	Microsite	Invasion	Fit	R^2	p-value	AIC	ΔAIC	Best fit
Precip × ANPP	open	high	linear	0.27	0.001	374.1	0.8	competitive
Precip × ANPP	open	high	poly	0.27	0.004	374.9		
Precip × ANPP	open	low	linear	0.63	0.002	106.1	1.3	competitive
Precip × ANPP	open	low	poly	0.61	0.009	107.4		
Precip × ANPP	shrub	high	linear	0.43	<0.001	427.2	1.6	competitive
Precip × ANPP	shrub	high	poly	0.422	<0.001	428.8		
Precip × ANPP	shrub	low	linear	0.15	0.200	90.7	-0.4	competitive
Precip × ANPP	shrub	low	poly	0.25	0.200	90.3		
Precip × PUE	open	high	linear	0.06	0.180	22.0	-0.8	competitive
Precip × PUE	open	high	poly	0.14	0.150	21.2		
Precip × PUE	open	low	linear	0.06	0.500	2.9	-0.3	competitive
Precip × PUE	open	low	poly	0.40	0.050	2.6		
Precip × PUE	shrub	high	linear	0.15	0.180	90.7	-0.4	competitive
Precip × PUE	shrub	high	poly	0.24	0.210	90.3		
Precip × PUE	shrub	low	linear	0.15	0.180	90.7	-0.4	competitive
Precip × PUE	shrub	low	poly	0.24	0.200	90.3		
Proportion invaded × ANPP	open	continuous covariate	linear	0.24	0.200	90.3	414.7	linear
Proportion invaded × ANPP	open	continuous covariate	poly	0.03	0.200	505.0		
Proportion invaded × ANPP	shrub	continuous covariate	linear	0.25	<0.001	536.6	0.2	competitive
Proportion invaded × ANPP	shrub	continuous covariate	poly	0.26	<0.001	536.8		
ANPP × Native abundance	shrub	NA	linear	0.33	<0.001	497.7	-4.9	poly
ANPP × Native abundance	shrub	NA	poly	0.40	<0.001	492.8		
ANPP × Native richness	shrub	NA	linear	0.05	0.080	127.5	1.8	competitive
ANPP × Native richness	shrub	NA	poly	0.03	0.200	129.3		

Table S2. Results of Moran's I test for spatial autocorrelation in aboveground net primary productivity (ANPP) across sites per study year. $P < 0.05$ indicates the presence of a year-specific positive spatial autocorrelation, i.e., ANPP was spatially clustered across our aridity gradient for that year.

Year	Moran's I	Expected	SD	P-value
2015	-0.033	-0.048	0.126	0.455
2016	0.592	-0.029	0.103	<0.001
2017	0.601	-0.029	0.103	<0.001
2019	-0.217	-0.077	0.140	0.841
2020	-0.004	-0.058	0.132	0.341

Table S3. Results of Moran's I test for temporal autocorrelation in aboveground net primary productivity (ANPP) within sites across study years. $P < 0.05$ indicates the presence of positive interannual autocorrelation, i.e., ANPP was temporally stable at that site (one instance – Carrizo 7, in **bold** for emphasis).

Site name	Moran's I	Expected	SD	P-value
Antelope Valley	-0.346	-0.200	0.232	0.736
Barstow 1	-0.590	-0.333	0.182	0.921
Barstow 2	-0.243	-0.333	0.292	0.378
Cal Nev Ari	NA	NA	NA	NA
Carrizo 1	-0.208	-0.200	0.156	0.520
Carrizo 2	-0.209	-0.200	0.163	0.522
Carrizo 3	-0.086	-0.200	0.142	0.213
Carrizo 4	-0.121	-0.200	0.154	0.304
Carrizo 5	-0.059	-0.200	0.160	0.184
Carrizo 6	-0.047	-0.200	0.171	0.185
**Carrizo 7**	**0.319**	**-0.111**	**0.186**	**0.010**
Cuyama 2	0.169	-0.143	0.318	0.163
Heart of the Mojave	NA	NA	NA	NA
Las Vegas	-0.072	-0.333	0.359	0.233
Mesquite	-0.300	-0.333	0.253	0.449
Panoche 1	-0.227	-0.200	0.228	0.548
Panoche 2	-0.059	-0.200	0.235	0.274
Panoche 3	-0.300	-0.200	0.221	0.675
Panoche Plateau	0.133	-0.143	0.308	0.185
Sheephole	-0.486	-0.333	0.113	0.912
Tecopa	-0.291	-0.333	0.266	0.436
Tejon	-0.116	-0.333	0.160	0.087
Yucca Grove	-0.413	-0.333	0.230	0.635
